# The Effects of Acute Aerobic Exercise on Blood Pressure, Arterial Function, and Heart Rate Variability in Men Living With HIV

**DOI:** 10.3389/fphys.2021.685306

**Published:** 2021-07-15

**Authors:** Juliana Pereira Barros, Tainah de Paula, Mauro Felippe Felix Mediano, Marcus Vinicius dos Santos Rangel, Walace Monteiro, Felipe Amorim da Cunha, Paulo Farinatti, Juliana Pereira Borges

**Affiliations:** ^1^Graduate Program in Exercise and Sports Sciences, Rio de Janeiro State University, Rio de Janeiro, Brazil; ^2^Department of Clinical Medicine, Rio de Janeiro State University, Rio de Janeiro, Brazil; ^3^Evandro Chagas National Institute of Infectious Diseases, Oswaldo Cruz Foundation, Rio de Janeiro, Brazil; ^4^Department of Research and Education, National Institute of Cardiology, Ministry of Health, Rio de Janeiro, Brazil; ^5^Graduate Program in Physical Activity Sciences, Salgado de Oliveira University, Niteroi, Brazil

**Keywords:** post-exercise hypotension, acquired immunodeficiency syndrome (AIDS), ambulatory blood pressure monitoring (ABPM), heart rate variability (HRV), autonomic nervous system (ANS), health

## Abstract

**Purpose:**

This study aims to investigate the effects of acute cycling on blood pressure (BP), arterial function, and heart rate variability (HRV) in men living with HIV (MLHIV) using combined antiretroviral therapy (cART).

**Methods:**

Twelve MLHIV (48.7 ± 9.2 years; 25.2 ± 2.8 kg m^–2^) and 13 healthy controls (41.2 ± 9.9 years; 26.3 ± 2.9 kg m^–2^) performed a cycling bout (ES) (intensity: 50% oxygen uptake reserve; duration: time to achieve 150 kcal—MLHIV: 24.1 ± 5.5 vs. controls: 23.1 ± 3.0 min; *p* = 0.45), and a 20-min non-exercise session (NES).

**Results:**

At rest (*p* < 0.05), MLHIV presented higher brachial systolic/diastolic BP (SBP/DBP: 123.2 ± 14.2/76.8 ± 6.3 vs. 114.3 ± 5.1/71.6 ± 2.6 mmHg) and central BP (cSBP/cDBP: 108.3 ± 9.3/76.5 ± 6.5 vs. 101.6 ± 4.9/71.3 ± 4.4 mmHg) vs. controls but lower absolute maximal oxygen uptake (2.1 ± 0.5 vs. 2.5 ± 0.3 L min^–1^) and HRV indices reflecting overall/vagal modulation (SDNN: 24.8 ± 7.1 vs. 42.9 ± 21.3 ms; rMSSD: 20.5 ± 8.5 vs. 38.1 ± 22.8 ms; pNN50: 3.6 ± 4.2 vs. 13.6 ± 11.3%). DBP postexercise lowered in controls vs. MLHIV (∼4 mmHg, *p* < 0.001; ES: 0.6). Moreover, controls vs. MLHIV had greater reductions (*p* < 0.05) in augmentation index (−13.6 ± 13.7 vs. −3.1 ± 7.2% min^–1^; ES: 2.4), and HRV indices up to 5 min (rMSSD: −111.8 ± 32.1 vs. −75.9 ± 22.2 ms min^–1^; ES: 3.8; pNN50: −76.3 ± 28.3 vs. −19.0 ± 13.7% min^–1^; ES: 4.4). Within-group (ES vs. NES; *p* < 0.05) reductions occurred in controls for SBP (∼10 mmHg, 2 h), DBP (∼6 mmHg, 20, 30, and 70 min), cSBP (∼9 mmHg, 30 min), cDBP (∼7 mmHg, 30 and 70 min), augmentation index (∼10%, 30 min), and pNN50 (∼20%; up to 2 h), while in MLHIV only cSBP (∼6 mmHg, 70 min) and cDBP (∼4 mmHg, 30 min) decreased. Similar increases (up to 5 min) in heart rate (∼22 bpm) and decreases in SDNN (∼18 ms) and rMSSD (∼20 ms) occurred in both groups.

**Conclusion:**

MLHIV under cART exhibited attenuated postexercise hypotension vs. healthy controls, which seemed to relate with impairments in vascular function.

## Introduction

The acquired immune deficiency syndrome (AIDS) caused by the human immunodeficiency virus (HIV) is a major public health issue. Up to 2020, 35 million people have died because of AIDS and 1.7 million were newly infected in 2019 (UNAIDS, 2020; World Health Organization, 2020). Although AIDS mortality has dramatically decreased since the introduction of combined antirretroviral therapy (cART), there is compelling evidence demonstrating that the HIV infection associated to prolonged cART increases the cardiovascular risk in people living with HIV (Feinstein et al., 2019).

On the other hand, it is well documented that regular physical exercise is capable of reducing cardiovascular risk and blood pressure levels (Pescatello et al., 2004a; Cornelissen and Fagard, 2005). The potential mechanisms of blood pressure decline due to exercise training seem to be linked to repeated reductions following single exercise bouts (Carpio-Rivera et al., 2016), which is referred to as postexercise hypotension (PEH) (Kenney and Seals, 1993). Although the mechanisms underlying PEH are not fully understood, it is accepted that this phenomenon results from a persistent drop in systemic vascular resistance induced by neural and vascular factors, which is not completely offset by increases in cardiac output (Halliwill et al., 2013).

People living with HIV usually present impaired autonomic modulation at rest (Glück et al., 2000; Neild et al., 2000; Correia et al., 2006; Lebech et al., 2007; Compostella et al., 2008) and after exercise (Borges et al., 2012). In addition, endothelial dysfunction has been described in this population (Lopes et al., 2019), even in early stages of HIV infection (Bush et al., 2019), which predisposes to increased arterial stiffness (Ferraioli et al., 2011; Anand et al., 2018). It is therefore feasible to suppose that blood pressure responses to acute exercise might be altered in those patients. We could find a single trial investigating this issue (Domingues et al., 2018), which failed to identify PEH after resistance exercise in women living with HIV. However, in what extent a single bout of aerobic exercise might induce blood pressure reduction among these patients is uncertain. A better understanding on this matter would be relevant to provide insights into supporting therapies counteracting cardiovascular damages induced by HIV infection and cART.

Given this gap in the literature, we aimed to investigate the effects of acute aerobic cycling exercise on blood pressure, arterial function, and cardiac autonomic modulation in men living with HIV (MLHIV) vs. age-matched non-infected counterparts. We hypothesized that PEH would be more likely to occur in healthy controls than among MLHIV.

## Materials and Methods

### Ethical Approval

All volunteers provided informed written consent before participation in the study, which complied with the recommendations laid on the Helsinki Declaration and gained approval from the Ethics Review Board of the Pedro Ernesto University Hospital (Rio de Janeiro, RJ, Brazil, CCAE 87616418.2.0000.5259).

### Subjects

Twelve MLHIV [age: 48.7 ± 9.2 years; body mass index (BMI): 25.3 ± 2.7 kg m^–2^] followed up at a tertiary-care university hospital, and 13 men without HIV/AIDS (controls) (age: 41.2 ± 9.9 years; BMI: 26.3 ± 2.9 kg m^–2^) were randomly recruited from the staff of the same institution to participate in this study. Eligible MLHIV should have been diagnosed with HIV/AIDS (Centers for Disease Control and Prevention, 1993) but should be asymptomatic and free from opportunist infections. Exclusion criteria were as follows: (a) use of cART for less than 6 months; (b) resting blood pressure ≥ 140/90 mmHg; (c) history of hypertension, coronary artery disease, ischemic disease, pulmonary disease, diabetes mellitus, Chagas disease, tuberculosis, or heart failure; (d) malnutrition; and (e) use of antidepressant or antihypertensive medication. Controls were screened for items b, c, d, and e.

### Experimental Design

The study was conducted during three visits to the laboratory, interspersed with 72-h intervals. Participants were instructed to avoid physical exercise in the 48 h and caffeine or alcohol in the 12 h prior to experimental sessions. All procedures took place at the same time of the day (7–8 a.m.) to minimize potential circadian effects on the outcomes, in a quiet temperature-controlled environment (21–22°C).

On the first visit, subjects underwent blood collection after 8 h fasting. After a light standardized breakfast, they were connected in supine position to an oxygen uptake (VO_2_) analyzer. The cuff for blood pressure measurement and belt for heart rate monitoring were positioned on the participant’s arm and chest, respectively. Brachial and central (aortic) blood pressure rates were measured after 30 min of rest, during which the heart rate variability (HRV) was assessed. Subsequently, a maximal cardiopulmonary exercise testing (CPET) was performed.

**FIGURE 1:**
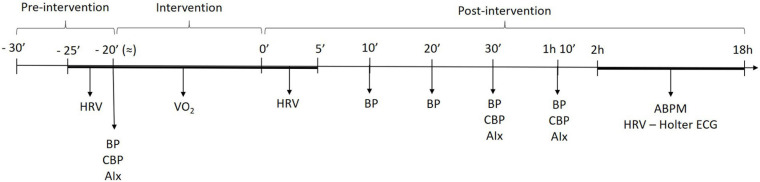
Timeline of measurements across submaximal exercise and non-exercise sessions. HRV, heart rate variability; BP, blood pressure; cBP, central blood pressure, AIx, augmentation index; ABPM, ambulatory blood pressure monitoring.

On second and third visits, non-exercise and aerobic exercise sessions were performed in a random counterbalanced order. Initially, the experimental setup of the first visit was mounted, with participants remaining at rest for 10 min. HRV, brachial, and central blood pressure assessments were repeated. Aerobic exercise sessions consisted of pedaling on cycle ergometer at intensity corresponding to 50% of oxygen uptake reserve (VO_2_R). The exercise went until energy expenditure of 150 kcal. In the non-exercise sessions, participants remained seated for 20 min mimicking the duration of the aerobic bout. Immediately after the experimental sessions, brachial blood pressure (10, 20, 30, and 70 min), central blood pressure, augmentation index (AIx) (30 and 70 min), and HRV (each 60 s up to 5 min) were assessed in supine position throughout 70-min recovery. The ambulatory blood pressure monitoring (ABPM) and Holter ECG System devices were placed 2 h after the end of the experimental sessions and returned 16 h later (next morning). Figure 1 summarizes the timeline of assessments during exercise and non-exercise sessions.

### Resting VO_2_ Assessment and Cardiopulmonary Exercise Testing

Breath-by-breath pulmonary gas exchanges were determined using a VO2000 analyzer (Medical Graphics^TM^, Saint Louis, MO, United States). Data were 30-s stationary time averaged, which provided a good compromise between removing noise while maintaining the underlying trend (Midgley et al., 2007). Prior to each test, the gas analyzers were calibrated according to the manufacturer’s instructions, using a certified standard mixture of oxygen (17.01%) and carbon dioxide (5.00%), balanced with nitrogen (AGA^TM^, Rio de Janeiro, RJ, Brazil). Ambient temperature and relative humidity ranged from 20 to 24°C and 50–70%, respectively. Resting and maximal VO_2_ were determined to calculate the percentage of VO_2_R, as described elsewhere (Fonseca et al., 2018; Cunha et al., 2020). The VO_2_ at rest was assessed following strict recommendations (Compher et al., 2006). Maximal CPET was performed on an electronic braked cycle ergometer (Cateye EC-1600, Cateye^TM^, Tokyo, Japan), using a ramp protocol designed to elicit maximal volitional effort within 8–12 min (Cunha et al., 2015a). Tests were considered maximal in the presence of at least three of the five following criteria (Howley et al., 1995): (a) maximum voluntary exhaustion; (b) ≥95% predicted maximal heart rate (HR) (220—age) or presence of heart rate (HR) plateau (ΔHR between two consecutive work rates ≤ 4 beats min^–1^); (c) presence of VO_2_ plateau (ΔVO_2_ between two consecutive work rates < 2.1 ml kg^–1^ min^–1^); (d) respiratory exchange ratio > 1.1; and (e) score of 10 on the Borg CR -10 scale.

### Submaximal Exercise Bout

Cycling bouts were performed at an intensity corresponding to 50% VO_2_R. The absolute VO_2_ corresponding to a given%VO_2_R was used to calculate the associated cycling power by applying the equation: VO_2_ cycling = 3.5 + 12.24 × power × body weight, where VO_2_ is in milliliters per kilogram per minute, power is in Watts, and body weight is in kilograms (American College of Sports Medicine, 2018). Cycling cadence was kept at 65 rpm, and the power output was adjusted whenever necessary to maintain the target intensity. The energy expenditure was calculated individually from the VO_2_ and VCO_2_ in liters per minute, using the Weir equation: Energy expenditure in kcal = [(3.941 × average VO_2_) + (1.106 × average VCO_2_)] × exercise time in minutes (Weir, 1949). The exercise bouts were terminated when participants achieved a total energy expenditure of 150 kcal, which represents the minimum threshold per session recommended by the ACSM to promote health (American College of Sports Medicine, 2018).

### Outcomes

#### Brachial Blood Pressure

At-office measurements of brachial blood pressure were performed in triplicate with 1-min intervals by the same trained professional, using a digital sphygmomanometer (Omron^TM^, HEM 7200, Matsusaka, Japan). ABPM was assessed on the non-dominant arm to obtain records from 2- to 18-h postinterventions (Welch Allyn model 6100, Poznań, Poland), every 20 min during daytime and every 30 min at night. Participants were instructed not to shower, perform physical exercise, or change their daily activities during the test, which was considered satisfactory when at least 70% of blood pressure readings were valid. All participants were given a standardized activity diary to register any unusual physical or emotional events. Patients were also asked to record the sleep and wake times during the recording.

#### Central (Aortic) Blood Pressure and Augmentation Index

Central blood pressure and AIx were assessed non-invasively by applanation tonometry, using the SphygmoCor System (AtCor Medical^TM^, Sydney, NSW, Australia). Radial artery waveforms were recorded from the radial artery at the wrist, and the sensor transmitting the pulse of the radial artery was placed over the radial artery for 10 s. The corresponding aortic waveforms were automatically generated from the radial artery waveform by a validated transfer function. The central blood pressure was computed from the radial artery pressure curve and calibrated with brachial blood pressure, as previously reported (Miyashita, 2012). Briefly, central augmentation pressure (AP) was calculated as the difference between the first and second systolic peaks on the central pressure waveform. The AIx—a measure of composite vascular function (Wilkinson et al., 1998, 2000; Ring et al., 2014)—was calculated as AP divided by central pulse pressure × 100 to give a percentage. The quality of the recordings was assured by discarding all SphygmoCor recordings with an operator index below 90.

#### Autonomic Modulation

Beat-to-beat HR was continuously recorded using a Polar RS800CX monitor (Polar Electro^TM^, Kempele, Finland), and signals were transferred to the Polar Precision Performance Software (Polar Electro, Kempele, Finland). After replacing the non-sinus beats by interpolated data derived from adjacent normal RR intervals, times series data were exported to a HRV analysis software (Kubios^TM^ HRV software, Biosignal Analysis and Medical Imaging Group, University of Kuopio, Kuopio, Finland). A Holter ECG system (CardioLight Digital^TM^, Cardio Sistema Ltda, São Paulo, SP, Brazil) was used to obtain HRV between 2- and 18-h postinterventions, through the CardioSmart^TM^ Institutional CS 550 software (Cardio Sistema Ltda, São Paulo, SP, Brazil).

In the present study, the following indices in time domain were assessed: standard deviation of the NN intervals (SDNN), square root of the mean squared successive differences from adjacent RR intervals (rMSSD), and percent number of pairs of adjacent RR intervals differing by more than 50 ms (pNN50). The SDNN reflects total variability, while rMSSD and pNN50 are estimates of short-term components of HRV reflecting the parasympathetic modulation (Task Force of the European Society of Cardiology and the North American Society of Pacing and Electrophysiology, 1996). HR recording and HRV analysis were performed as previously recommended (Task Force of the European Society of Cardiology and the North American Society of Pacing and Electrophysiology, 1996; Bourdillon et al., 2017; Shaffer and Ginsberg, 2017). All devices were installed by the same trained professional, and data were analyzed on a single computer.

### Statistical Analysis

A total of eight individuals in each group was estimated as necessary, according to sample size *a priori* calculations performed using the G^∗^Power^TM^ 3.0.10 software (Kiel University, Kiel, Germany) considering 80% power, 5% significance level, and effect size of 0.44 based on acute exercise-induced change in blood pressure of −3.1 mmHg (Carpio-Rivera et al., 2016). Data normality was ratified by Shapiro–Wilk statistics, and therefore data were expressed as mean ± standard deviation.

Differences between MLHIV and controls at baseline were tested by unpaired *t*-tests. Linear mixed models adjusted for baseline values were fitted to evaluate the effects of exercise on changes from baseline in MLHIV and controls. The following approaches were adopted: (a) within-between group analysis, with models including group, time, session (non-exercise or aerobic exercise) as fixed effects and group × time × session interaction (power: 60%); (b) within-group analysis, with models including time and session (non-exercise or aerobic exercise) as fixed effects, and time × session interaction (power: 76%). The adjustment of models were evaluated based on Bosker/Snijders *R*-squared values (Recchia, 2010), and rate of changes between sessions was expressed by β coefficients. Additionally, Cohen’s *d* effect sizes (ES) were calculated for significant differences between sessions.

Due to the probable insufficient statistical power of the three-way interaction model, between-group analysis was complemented by comparing the areas under the curves (AUCs) of exercise net effects [(post-pre-exercise session) - (post-pre-non-exercise session)] on outcomes in MLHIV and controls, using unpaired *t*-tests. In all cases, statistical analyses were performed using the Stata 13.0 software (StataCorp, College Station, TX, United States), and significance level was fixed at *p* ≤ 0.05.

## Results

### Baseline Sample Characteristics and Submaximal Exercise Bouts

Clinical, cardiovascular, and autonomic variables at rest are presented in Table 1. No difference was detected between groups for age, height, body mass, body mass index, abdominal circumference, LDL cholesterol, triglycerides, relative maximal VO_2_, HR at rest, and AIx. However, MLHIV presented higher glucose and lower absolute maximum VO_2_, total cholesterol, and HDL cholesterol than controls. As for cardiovascular and autonomic outcomes, MLHIV exhibited higher brachial and central blood pressure and lower SDNN, rMSSD, and pNN50 than controls. Moreover, 66% of patients were using nucleoside reverse transcriptase inhibitors, 66% non-nucleoside reverse transcriptase inhibitors, 41% protease inhibitors, and 25% integrase inhibitors.

**TABLE 1:** Clinical, cardiovascular, and autonomic parameters at rest in controls and men living with HIV (MLHIV).

	Controls (*n* = 13)	MLHIV (*n* = 12)	*p*-value*
**Clinical parameters**			
Age (years)	41.2 (9.9)	48.7 (9.2)	0.07
Height (cm)	177.9 (4.9)	178.6 (5.4)	0.75
Body mass (kg)	83.4 (11.9)	80.9 (11.2)	0.59
Body mass index (kg m^–2^)	26.3 (2.9)	25.2 (2.8)	0.39
Abdominal circumference (cm)	90.1 (9.1)	92.3 (9.3)	0.60
Glucose (mg dl^–1^)	89.4 (9.8)	97.2 (6.2)	**0.05**
Total cholesterol (mg dl^–1^)	205.2 (34.7)	170.6 (28.7)	**<0.01**
LDL cholesterol (mg dl^–1^)	126.2 (29.1)	107.5 (28.9)	0.12
HDL cholesterol (mg dl^–1^)	52.5 (10.8)	37.5 (7.9)	**<0.01**
Triglycerides (mg dl^–1^)	132.6 (81.8)	180.5 (86.7)	0.17
Maximal oxygen uptake (L min^–1^)	2.5 (0.3)	2.1 (0.5)	**0.03**
Maximal oxygen uptake (ml kg^–1^ min^–1^)	30.4 (6.1)	26.4 (4.3)	0.07
Years diagnosed with HIV	–	17.3 (6.6)	–
Years taking cART	–	17.1 (7.4)	–
T CD4 (cell mm^–3^)	–	683.6 (271.7)	–
T CD8 (cell mm^–3^)	–	857.2 (419.8)	–
Undetectable viral load (*n*, %)	–	12 (100)	–
**Cardiovascular and autonomic parameters**			
Heart rate (bpm)	64.9 (8.8)	71.2 (12.5)	0.15
Systolic blood pressure (mmHg)	114.3 (5.1)	123.2 (14.2)	**0.04**
Diastolic blood pressure (mmHg)	71.6 (2.6)	76.8 (6.3)	**0.01**
Central systolic blood pressure (mmHg)	101.6 (4.9)	108.3 (9.3)	**0.03**
Central diastolic blood pressure (mmHg)	71.3 (4.4)	76.5 (6.5)	**0.03**
Augmentation index (%)	14.3 (10.7)	15.2 (12.3)	0.85
SDNN (ms)	42.9 (21.3)	24.8 (7.1)	**0.01**
rMSSD (ms)	38.1 (22.8)	20.5 (8.5)	**0.02**
pNN50 (%)	13.6 (11.3)	3.6 (4.2)	**0.01**

*Student *t*-test. Data expressed as mean (SD). *cART*, combined antiretroviral therapy; *SDNN*, standard deviation of normal to normal intervals; *rMSSD*, root mean square of successive differences between normal intervals; *pNN50*, percentage of differences between adjacent normal intervals. *p*-values in bold denote statistical significant differences.

Table 2 depicts data for total duration, HR, and VO_2_ elicited by the acute exercise bouts, which were similar between groups.

**TABLE 2:** Characteristics of submaximal exercise session in controls and men living with HIV (MLHIV).

	Controls (*n* = 13)	MLHIV (*n* = 12)	*p*-value*
Duration (min)	23.2 (3.1)	24.9 (5.6)	0.45
Heart rate (bpm)	122.7 (10.2)	125.3 (14.4)	0.61
Oxygen uptake (% reserve)	53.2 (3.9)	55.7 (4.9)	0.19

*Student *t*-test. Data expressed as mean (SD).

### Acute Effects of Submaximal Aerobic Exercise

Residual plots for all models were visually examined and did not demonstrate deviations from the regression assumptions. As expected, the linear model including group as fixed effect (three-way interaction) lacked significance for all outcomes (*p* ≥ 0.08), while the approach including time and session proved to be significant (*p* ≤ 0.03). Bosker/Snijders *R*-squared for within-group models discriminated by outcome were always non-negative (controls: 0.13–0.85; MLHIV: 0.45–0.96), indicating low chances of misspecification giving the explanatory variables added to the models. Detailed *R*-squared data per outcome and group are presented in Supplementary Table 1.

#### Brachial Blood Pressure

**FIGURE 2:**
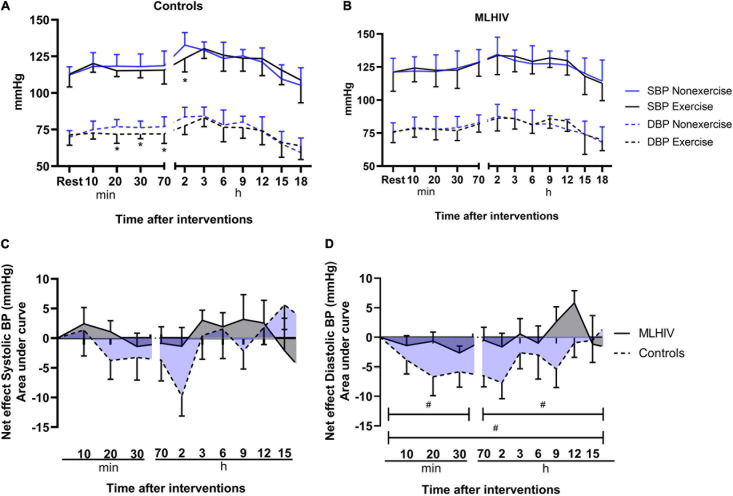
Blood pressure after submaximal exercise or non-exercise sessions in controls **(A)** and men living with HIV **(B)** and exercise net effects on systolic **(C)** and diastolic **(D)** blood pressure. SBP, systolic blood pressure; DBP, diastolic blood pressure. *p < 0.05 for changes from baseline (exercise vs. non-exercise session) using linear mixed models. ^#^*p* < 0.05 for differences between areas under the curves of controls vs. MLHIV.

Resting blood pressure measured on the first visit and before the experimental conditions (exercise and non-exercise) was similar in controls (114.3/71.6 vs. 112.3/69.9 vs. 112.9/71.4 mmHg, respectively; *p* > 0.38) and MLHIV (123.2/76.8 vs. 121.2/75.5 vs. 121.1/76.0 mmHg, respectively; *p* > 0.59). Figure 2 presents absolute values of at-office and ambulatory brachial blood pressure after the experimental sessions in controls (Figure 2A) and MLHIV (Figure 2B), and exercise net effects on systolic blood pressure (SBP, Figure 2C) and diastolic blood pressure (DBP, Figure 2D). In both groups, no difference between sessions was detected for SBP, except for controls that presented lower values 2 h postexercise vs. non-exercise sessions (123.6 ± 9.3 vs. 132.7 ± 8.7 mmHg; β = −9.73; 95% CI = −18.6 to −0.8; *p* = 0.03; ES: 1.5). Controls showed lower DBP after 20 min (115.2 ± 4.0 vs. 118.3 ± 8.0 mmHg; β = −6.69; 95% CI = −11.4 to −1.9; *p* < 0.01; ES: 0.55), 30 min (115.4 ± 5.0 vs. 118.0 ± 8.6 mmHg; β = −5.84; 95% CI = −10.5 to −1.1; *p* = 0.01; ES: 0.80), and 70 min (115.6 ± 9.5 vs. 118.6 ± 10.1 mmHg; β = −6.53; 95% CI = −11.2 to −1.7; *p* < 0.01; ES: 1.3) after exercise vs. non-exercise sessions, while no difference was detected for MLHIV.

There was no difference between controls and MLHIV with regard to AUCs of exercise net effects on SBP (Figure 2C) (overall: −11.3 ± 23.0 vs. −4.5 ± 12.8 mmHg min^–1^; *p* = 0.37). On the other hand, greater reductions in DBP (Figure 2D) were found in controls vs. MLHIV along the first 70 min of recovery (−19.6 ± 12.5 vs. −5.0 ± 7.0 mmHg min^–1^; *p* < 0.01; ES: 0.7), ABPM (−16.0 ± 18.1 vs. 7.6 ± 6.8 mmHg min^–1^; *p* < 0.001; ES: 0.4), and total follow-up (−42.8 ± 22.8 vs. 0.9 ± 11.3 mmHg min^–1^; *p* < 0.0001; ES: 0.6). This corresponded to an overall average difference of −4 mmHg between groups.

#### Central Blood Pressure and Augmentation Index

**FIGURE 3:**
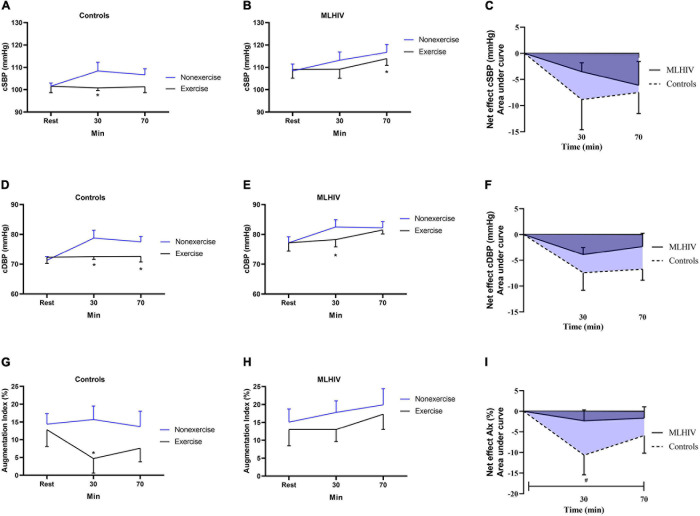
Absolute values and exercise net effects of central systolic blood pressure **(A–C)**, central diastolic blood pressure **(D–F)**, and augmentation index **(G–I)** after exercise and non-exercise sessions in controls and men living with HIV (MLHIV). cSBP, central systolic blood pressure; cDBP, central diastolic blood pressure. *p < 0.05 for changes from baseline (exercise vs. non-exercise session) using linear mixed models. ^#^*p* < 0.05 for differences between areas under the curves of controls vs. MLHIV.

Figure 3 depicts absolute values and exercise net effects on central (aortic) blood pressure (Figures 3A–F) and AIx (Figures 3G–I) in controls and MLHIV. In the within-group analysis, controls had lower cSBP at 30 min (100.8 ± 4.3 vs. 108.4 ± 13.6 mmHg; β = −8.21; 95% CI = −15.6 to −0.7; *p* = 0.03; ES: 0.7), and lower cDBP at 30 min (72.5 ± 3.3 vs. 78, 8 ± 9.0 mmHg; β = −7.58; 95% CI = −12.6 to −2.4; *p* < 0.01; ES: 0.8), and 70 min (72.6 ± 6.8 vs. 77.5 ± 6.3 mmHg; β = −6.18; 95% CI = −11.2 to −1.0*; p* = 0.01; ES: 2.0) after exercise vs. non-exercise sessions. In MLHIV, reductions in postexercise vs. non-exercise sessions were detected for cSBP at 70 min (116.7 ± 11.1 vs. 113.9 ± 9.4 mmHg; β = −6.53; 95% CI = −12.6 to −0.3; *p* = 0.03; ES: 0.3) and cDBP at 30 min (78.3 ± 8.1 vs. 82.5 ± 7.7 mmHg; β = −4.20; 95% CI = −7.6 to −0.7; *p* = 0.01; ES: 0.95). Lower AIx was found for controls at 30 min postexercise vs. non-exercise sessions (4.7 ± 14.7 vs. 15.7 ± 13.1%; β = −10.53; 95% CI = −18.9 to −2.0; *p* = 0.01; ES: 1.0), while no difference between sessions occurred for MLHIV.

No statistical difference between controls and MLHIV occurred for AUCs of exercise net effects on cSBP (−12.5 ± 15.7 vs. −6.5 ± 7.8 mmHg min^–1^; *p* = 0.24) and cDBP (−10.7 ± 9.0 vs. −5.0 ± 4.9 mmHg min^–1^; *p* = 0.06). On the other hand, the AIx reduction was greater in controls vs. MLHIV (−13.6 ± 13.7 vs. −3.1 ± 7.2% min^–1^; *p* = 0.02; ES: 2.4).

#### Heart Rate and Heart Rate Variability

**FIGURE 4:**
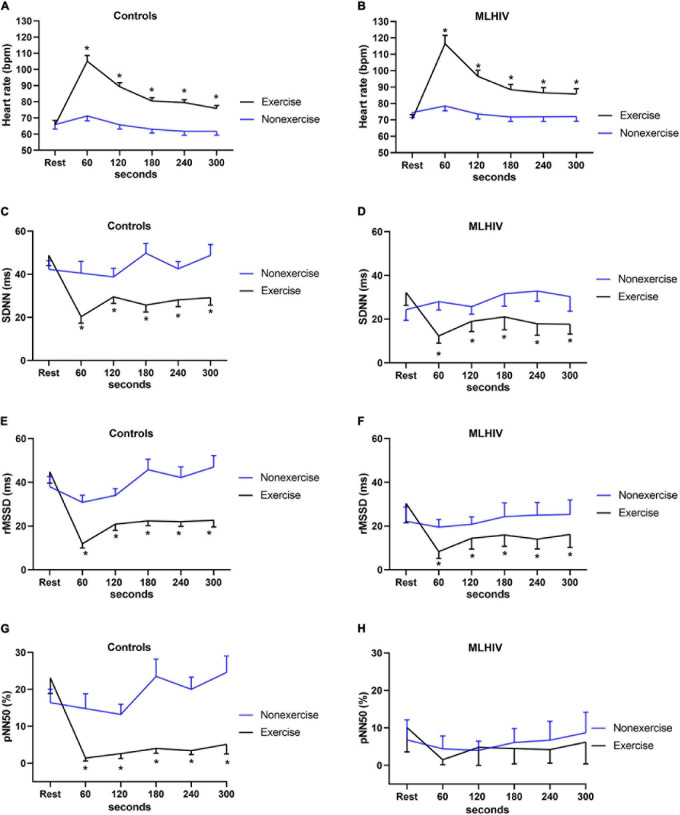
Heart rate **(A,B)** and heart rate variability indices **(C–H)** from baseline to 5 min after exercise or non-exercise sessions in controls and men living with HIV (MLHIV). SDNN, standard deviation of normal to normal intervals; rMSSD, root mean square of successive differences between normal intervals; pNN50, percentage of differences between adjacent normal intervals. *p < 0.05 for changes from baseline (exercise vs. non-exercise session) using linear mixed models.

**FIGURE 5:**
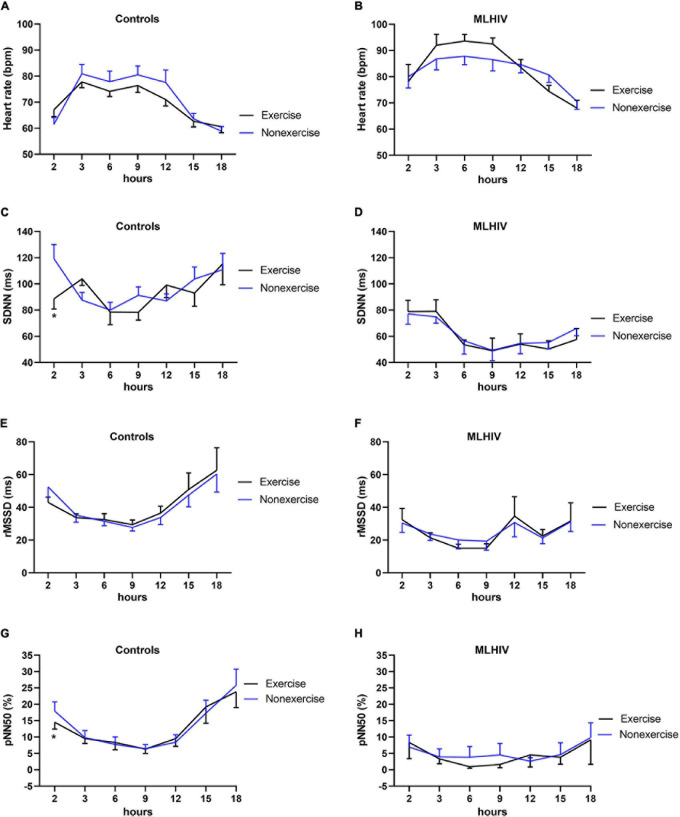
Heart rate **(A,B)** and heart rate variability indices **(C–H)** during 18 h ambulatory blood pressure monitoring after exercise or non-exercise sessions in controls and men living with HIV (MLHIV). SDNN, standard deviation of normal to normal intervals; rMSSD, root mean square of successive differences between normal intervals; pNN50, percentage of differences between adjacent normal intervals. *p < 0.05 for changes from baseline (exercise vs. non-exercise session) using linear mixed models.

Figure 4 shows HR and HRV data from baseline up to 5 min (300 s) following the experimental sessions. Both groups presented higher HR and lower SDNN and rMSSD in all time points after exercise vs. non-exercise sessions, but only controls exhibited lower pNN50. Figure 5 presents absolute values for HR and HRV between 2 and 18 h after the experimental conditions. In both groups, HR, SDNN, and rMSSD were similar in exercise and non-exercise sessions. The only exception was the lower pNN50 in controls vs. MLHIV after 2 h postexercise vs. non-exercise sessions (14.5 ± 7.2 vs. 17.9 ± 9.4%; β = −11.27; 95% CI = −20.5 to −1.9; *p* = 0.01; ES: 0.4).

**FIGURE 6:**
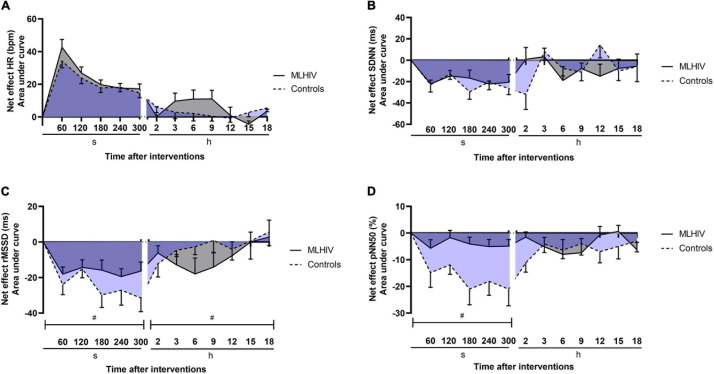
Exercise net effects on heart rate **(A)** and heart rate variability indices **(B–D)** in controls and men living with HIV (MLHIV). SDNN, standard deviation of normal to normal intervals; rMSSD, root mean square of successive differences between normal intervals; pNN50, percentage of differences between adjacent normal intervals. ^#^*p* < 0.05 for differences between areas under the curves of controls vs. MLHIV.

Exercise net effects for HR and HRV are presented in Figure 6. Comparisons of AUCs in the first 300 s revealed that controls had greater reductions vs. MLHIV in rMSSD (−111.8 ± 32.1 vs. −75.9 ± 22.2 ms min^–1^; *p* < 0.01; ES: 3.8) and pNN50 (−76.3 ± 28.3 vs. −19.0 ± 13.7% min^–1^; *p* < 0.0001; ES: 4.4). On the other hand, the Holter analysis showed greater rMSDD reduction postexercise in MLHIV than controls (−45.7 ± 26.1 vs. −13.6 ± 24.5 ms min^–1^; *p* < 0.01; ES: 0.7).

## Discussion

The present study compared the blood pressure, vascular function, and cardiac autonomic modulation after exercise and non-exercise sessions in MLHIV and non-infected controls. To the best of our knowledge, this is the first controlled trial describing cardiovascular responses to acute aerobic exercise in MLHIV, since prior studies addressing vascular function in these patients reported only data at rest (Leite et al., 2017). The major finding was that PEH was attenuated in MLHIV vs. healthy controls—while reductions after exercise have been detected in brachial diastolic blood pressure in controls, changes did not occur in MLHIV. Postexercise decreases in AIx and HRV markers reflecting vagal modulation were also greater in controls vs. MLHIV.

Our data concur with a prior study (Domingues et al., 2018) that failed to identify PEH after resistance exercise in women living with HIV. In that study, five responders out of 12 patients (decreases in SBP > 4 mmHg) had lower CD4/CD8 ratio and used cART for shorter periods. This suggests that the attenuated PEH presently observed in MLHIV might have been influenced by the prolonged use of cART. The average blood pressure reduction in the first 2 h of postexercise recovery was approximately 3.0/4.5 mmHg (SBP/DBP) in controls, which is consistent with values reported for individuals with normal blood pressure (∼4.5/2.6 mmHg) (Perrier-Melo et al., 2020) and higher than decreases in MLHIV (∼0/1 mmHg). Prior research has suggested that the length and magnitude of postexercise hypotension may be influenced by exercise session characteristics, such as duration, intensity, or volume (Brito et al., 2018; Fonseca et al., 2018). Therefore, at least in theory, longer (>25 min) and more intense (>50% of VO_2_R) exercise would elicit greater hypotensive responses (de Brito et al., 2019). Our exercise protocol was defined based on recommendations from the ACSM for minimum energy expenditure during health-oriented exercise sessions (American College of Sports Medicine, 2018). Nevertheless, from a clinical perspective, epidemiological studies indicate that a decrease of 2 to 5 mmHg in SBP could reduce the mortality due to cardiovascular causes by 6–14% (Carpio-Rivera et al., 2016).

An important aspect of our study is the inclusion of a non-exercise session to control time effects on blood pressure (de Brito et al., 2019). Due to the circadian variation, blood pressure increases progressively in the morning before showing a decrease (Hermida et al., 2007). This is consistent with our data from non-exercise sessions in both groups. Apparently, the exercise session lowered this circadian effect on blood pressure in controls, as previously reported (Cucato et al., 2015; de Brito et al., 2015; Queiroz et al., 2017). It is also worth mentioning that the exercise bouts have been matched for the overall energy expenditure, therefore negating the influence of exercise volume on the magnitude and duration of acute blood pressure reduction (Jones et al., 2007; Fonseca et al., 2018). This strategy resulted from the premise that central baroreflex plays an important role in eliciting PEH. During exercise, vascular smooth muscle (myogenic tone) (Phan et al., 2021) and muscle afferent fibers (exercise pressor reflex) contribute to reset the blood pressure to a higher level (Halliwill et al., 2013). When exercise is terminated, a decrease in sympathetic activity resets the baroreflex to a lower level, contributing to the acute blood pressure reduction (Chen and Bonham, 2010; Halliwill et al., 2013). A greater amount of muscle work—in other words, exercise volume—increases the exercise pressor response (Halliwill et al., 2013). Thus, it is feasible to speculate that the exercise volume would be a major determinant of the PEH phenomenon.

Several studies investigating PEH did not match exercise sessions performed with different intensity for the total amount of work (Forjaz et al., 2004; Pescatello et al., 2004b; Eicher et al., 2010; Casonatto et al., 2011), and this helps on explaining why one of them failed to detect hypotensive responses (Casonatto et al., 2011), while others claimed that intensity would be more determinant than duration to produce PEH (Forjaz et al., 2004; Pescatello et al., 2004b; Eicher et al., 2010). Trials assessing the blood pressure after acute aerobic exercise bouts performed with different intensities, but similar volume (energy expenditure, time × intensity, etc.) have consistently reported similar hypotensive effects (Jones et al., 2007; Fonseca et al., 2018; Cunha et al., 2020).

It is well accepted that PEH is more likely to occur in individuals with high than normal blood pressure (Brito et al., 2014). However, although MLHIV presented higher brachial and central blood pressure at rest than controls, in both groups, those outcomes felt within the normal range (Reboussin et al., 2018). According to the American Heart Association, studies are inconsistent on whether the prevalence of hypertension is higher in patients with treated HIV vs. uninfected individuals (Feinstein et al., 2019). However, less controversial is the association between HIV infection and autonomic dysfunction—the accumulated evidence suggests a shift toward sympathetic dominance (McIntosh, 2016), which concurs with our data.

Besides predisposing patients to higher cardiovascular risk (McIntosh, 2016), autonomic dysfunction also seem to influence parasympathetic reactivation after exercise (Cunha et al., 2015b). In this sense, Cunha et al. (2015b) reported that individuals with lower vagal modulation at rest tend to exhibit slower postexercise parasympathetic reactivation. We could not confirm a delayed vagal reactivation and sympathetic withdrawal within 5 min of recovery after the exercise performed by MLHIV, but rather an attenuated vagal withdrawal. Borges et al. (2012) observed that people living with HIV exhibited lower vagal modulation during the first 30 min of postexercise recovery in comparison with healthy controls. However, since the vagal modulation at rest was already different between groups, and no data have been provided demonstrating in what extent vagal modulation was reactivated in comparison with baseline, assumptions on how fast vagal reactivation and sympathetic withdrawal occurred after exercise could not be made. Our results indicate that the time course of autonomic responses during recovery did not affect the effects of acute exercise on blood pressure. However, the contribution of autonomic dysfunction in precluding the occurrence of PEH among MLHIV cannot be discarded, since only the parasympathetic modulation has been indirectly assessed. Further research is warranted to confirm these findings, including direct assessments of both sympathetic and parasympathetic activities.

The role of changes in cardiac autonomic control to produce PEH remains controversial even among uninfected individuals. While some studies reported a reduction in sympathetic activity associated with increased vagal activity (Park et al., 2006), others reported no changes (Park et al., 2008; Anunciacao et al., 2016) or observed increased sympathetic activity (Teixeira et al., 2011; Cunha et al., 2016). It has been suggested that an increase in sympathetic outflow concomitant to PEH would be a reflex response to counteract the reduction in blood pressure and the baroreflex resetting (MacDonald, 2002). Our findings partially concur with this premise, since during postexercise recovery the HR was higher and indices reflecting vagal modulation were lower vs. pre-exercise in both MLHIV and controls. Moreover, the greater decrease in DBP was concomitant with lower RMSSD and pNN50 in controls vs. MLHIV. It is therefore feasible to speculate that PEH among controls was not mediated by increased vagal activity (or by opposition, lowered sympathetic activity). In this case, the hypotensive response to exercise would rely on the ability of local vasodilator mechanisms to override the effects of sympathetic activation (Fonseca et al., 2018). This is again in agreement with our results in regards to AIx. Acute reductions in sympathetic vasoconstrictor activity have been reported in exercising muscles (i.e., functional sympatholysis) (Moynes et al., 2013). This phenomenon is thought to be mediated by locally released substances that modulate the effect of noradrenaline on α-receptors, such as histamine, opioids, nitric oxide, prostaglandins, or ATP (Halliwill, 2001), which are yet to be properly assessed in MLHIV.

There is strong accumulated evidence indicating a decrease in arterial stiffness following acute aerobic exercise (Mutter et al., 2017; Pierce et al., 2018). Arterial stiffness depends on several factors, such as endothelial function, smooth muscular vascular tone, and structural features (Martinez-Ayala et al., 2020). It has been proposed that a relaxation of vascular smooth muscle transfers stress from the less extensive collagen fibers to elastin, which could partially account for decreases in arterial stiffness after exercise (Mutter et al., 2017). Evidence demonstrates that changes in immune activity due to HIV infection may increase the pulse wave velocity (Boccara et al., 2006; Rider et al., 2014), disrupting the activity of the matrix metalloproteinase (MMPs) (Misse et al., 2001) and degrading collagen, elastin, laminin, and fibrillin within the arterial wall (Martinez-Ayala et al., 2020). The consequent increasing in vascular resistance limits the vasodilation response during exercise. Accordingly, in the present study, greater postexercise reduction in AIx was found in controls vs. MLHIV. This is suggestive that vascular mechanisms could partially explain the PEH detected in controls, but not in MLHIV.

The major limitation of the present study was the lack of data regarding additional hemodynamic outcomes (e.g., stroke volume, cardiac output, and peripheral resistance), which precluded further analysis on whether the attenuated PEH in MLHIV resulted from central or peripheral mechanisms. Second, despite being compatible with health-oriented exercise prescription (American College of Sports Medicine, 2018), the exercise bout was of relatively short duration and moderate intensity, which limits the generalization of our findings to exercise settings with greater volume (vigorous intensity and/or longer duration). Another important feature refers to the relatively small sample of men only. The small sample probably contributed to the lack of significance of the linear mixed model including “group” as fixed effect, which would be the optimal approach. The exclusive participation of men in the study limits its external validity. However, the inclusion of women might introduce a confounding factor due to differences in sex hormones affecting the autonomic nervous system and blood pressure responses to acute exercise (Carpio-Rivera et al., 2016).

## Conclusion

An aerobic cycling bout performed with moderate intensity and relatively short duration seemed to be capable to induce PEH in non-infected controls, but not in MLHIV using cART. Although MLHIV presented autonomic dysfunction at rest, no evidence was found of delayed vagal reactivation and sympathetic withdrawal within 5 min after exercise in this group. The acute reduction in DBP among controls was concomitant with greater postexercise decreases in HRV indices reflecting vagal modulation vs. MLHIV. Although no changes between groups were detected for central blood pressure, controls exhibited greater reductions in AIx after exercise than MLHIV.

Overall, these data are indicative of the role of vascular responses to produce PEH, and that the attenuated postexercise blood pressure reduction in MLHIV may have resulted from vascular dysfunction limiting vasodilation. In practical terms, our findings suggest that aerobic exercise sessions with appropriate volume may contribute to reduce blood pressure and cardiovascular risk in MLHIV. However, further studies investigating the effects of acute exercise performed with different intensities and durations on cardiovascular responses in people living with HIV under cART are warranted, to provide information to optimize exercise prescription aiming to reduce blood pressure, improve autonomic control, and prevent vascular dysfunction in those patients.

## Data Availability Statement

The raw data supporting the conclusions of this article will be made available by the authors, without undue reservation.

## Ethics Statement

The studies involving human participants were reviewed and approved by Ethics Review Board of the Pedro Ernesto University Hospital/Rio de Janeiro State University. The patients/participants provided their written informed consent to participate in this study.

## Author Contributions

JBa, TP, WM, FC, PF, and JBo were involved in the conception and design of the research. JBa, TP, and MR collected the data. JBo and MM analyzed the data. JBa, JBo, and PF drafted the manuscript. All authors revised, edited, and approved the final manuscript.

## Conflict of Interest

The authors declare that the research was conducted in the absence of any commercial or financial relationships that could be construed as a potential conflict of interest.
