# Supplementary material for: The Effects of Acute Aerobic Exercise on Blood Pressure, Arterial Function, and Heart Rate Variability in Men Living With HIV
Source: Front Physiol. 2021 Jul 15;12:685306. doi: 10.3389/fphys.2021.685306 (PMC8320391; doi:10.3389/fphys.2021.685306)
Supplement: Supplementary file 1 [file Table_1.DOCX]

**Supplementary Table 1.** Bosker/Snijders R-squared for linear mixed model analysis in Controls and Men Living With HIV (MLHIV).

|  | Controls  (n = 13) | MLHIV  (n = 12) |
| --- | --- | --- |
| Brachial systolic blood pressure | 0.42 | 0.96 |
| Brachial diastolic blood pressure | 0.62 | 0.96 |
| Ambulatory systolic blood pressure | 0.19 | 0.57 |
| Ambulatory diastolic blood pressure | 0.13 | 0.45 |
| Central systolic blood pressure | 0.35 | 0.93 |
| Central diastolic blood pressure | 0.58 | 0.96 |
| Augmentation index | 0.75 | 0.96 |
| Heart rate within 5 min | 0.76 | 0.79 |
| SDNN within 5 min | 0.39 | 0.92 |
| rMSSD within 5 min | 0.68 | 0.96 |
| pNN50 within 5 min | 0.77 | 0.98 |
| Heart rate over 18 h | 0.66 | 0.64 |
| SDNN over 18 h | 0.33 | 0.72 |
| rMSSD over 18 h | 0.67 | 0.83 |
| pNN50 over 18 h | 0.85 | 0.94 |

SDNN, standard deviation of normal to normal intervals; rMSSD, root mean square of successive differences between normal intervals; pNN50, percentage of differences between adjacent normal intervals.
